# Links between Adolescent Athletes’ Prosocial Behavior and Relationship with Parents: A Mixed Methods Study

**DOI:** 10.3390/sports6010004

**Published:** 2018-01-17

**Authors:** Aušra Lisinskienė, Marc Lochbaum

**Affiliations:** 1 Department of Theory of Sports, Lithuanian University of Educational Sciences, Vilnius 08106, Lithuania; 2 Department of Kinesiology and Sport Management, Texas Tech University, Lubbock, TX 79409-3011, USA; marc.lochbaum@ttu.edu

**Keywords:** youth sport, parents, adolescents, prosocial behavior, mixed methods

## Abstract

Adolescent relationships with parents are of the highest importance. The relationships likely reflect the nature of internal working models in youth sport that may well function as a psychological template during the construction of youth prosocial behavior. However, researchers’ focus to date has concerned specific aspects of parental practices in child-based sporting activities. There is a lack of research covering parent-athlete interpersonal relationships concerned with how the relationships affect adolescent prosocial behavior. The purpose of this mixed methods explanatory sequential study was to examine teenage athletes’ prosocial behavior and their relationships with parents. To achieve our purpose, we obtained quantitative data from 1348 athletes and non-athletes (ages 12–16), and qualitative data from 12 adolescent athletes and 12 youth sports parents. In the quantitative phase, we assessed adolescent prosocial behavior regarding the following six forms of prosocial behavior: public, anonymous, dire, compliant, altruistic and emotional. In the qualitative follow-up, three themes emerged from the adolescent athlete’s perspective: (1) sport as an escape; (2) parent-child relationships in youth sports; (3) adolescents’ desired behavior. Three themes emerged from the parental perspective: (1) sport as protection and as a school of life; (2) painful decisions to release a child; (3) understanding adolescent behavior. We found protection from delinquent behavior and increased prosocial behavior with securely attached young athletes who are actively involved in sports.

## 1. Introduction

Recent decades have seen a growing concern for the prospects of today’s youth. The significant problems with young people relate to pervasive, destructive and inappropriate behavior: delinquency, aggression, bullying, drug abuse, etc. [1,2]. These problems are the result of changing social factors: both parents work full-time, single-parent families, and unattended children at home [1]. Successful personality development depends on some external factors: family, school, positive role models, purposefully and deliberately chosen ways of self-realization and leisure time activities [3].

Regarding leisure activities, investigations of adolescent involvement in sports activities are worth conducting. An analysis of children’s and especially adolescents’ behavior draws attention to the role of sports in the development of prosocial behavior [4]. Society considers playing sports an attractive social activity with a good image and a positive effect on a child’s development [5]. Also, the role of the family in a child’s positive personality development through sports is particularly highlighted [3,6]. Close and stable relationships between children and parents ensure consistent feelings of security and confidence [7]. Affectionate relationships between parents and children influence the formation of behavior [6,8]. In this context, individual understanding behavior, especially prosocial behaviors (i.e., actions such as comforting, sharing, and volunteerism that benefit others), is especially intriguing. Such acts are desirable and beneficial to society [9].

Researchers define prosocial behavior as a voluntary behavior intended to help or benefit another [9,10]. Thus, researchers foster the recognition that knowledge of prosocial behavior, as an essential phenomenon, can be useful for a better understanding of overall psychosocial development during adolescence [11]. Substantial evidence supports prosocial behavior learned through observation and verbal expression. We grounded our study on Social Cognitive Theory [12], which is one of the most widely used models to address the importance of observational learning, imitation, and modeling. Social Cognitive Theory emphasizes learning from the social environment and postulates reciprocal interactions among personal, behavioral, and social/environmental factors. Sport as a social environment plays an essential role in the lives of many adolescents [13].

Many researchers have emphasized that the sporting environment promotes the prosocial development of youth [4,14,15] where new friends emerge, further contacts are established, and adolescents become part of a growing social network. Sporting activities encourage children to help others and to develop altruism and empathy [16]. Adolescents who play sports are friendlier to peers, especially to those involved in similar activities, and have less contact with peers who are inclined to engage in problematic behavior [13,17].

However, both positive and negative associations exist with sports participation, mainly due to its competitive nature and the excessive pressure to win. Researchers and most likely most countries consider aggression a significant observable problem in sporting events from the level of youth sports to games played at the highest levels of competition [18]. Furthermore, the professionalization of sports has led to increasing pressure on competition outcomes, including the “winning at all costs” mentality. This mentality promotes substance abuse, reduced competition fairness, and the depreciation of positive societal values [14]. Therefore, understanding moral, prosocial behavior relative to youth participation in organized sports is an essential area of research.

As reviewed, past research explored the differences in prosocial behavior between genders, the influence of genetic inheritance, and the relationship between personal characteristics and psychological factors. However, research on environmental factors, such as the influence of parents on adolescent prosocial behavior, the sporting environment and the effects of the interactions of these factors, is limited. We intended to explain the link between teenage athletes’ prosocial behavior and their relationships with parents. This study used a sports context to analyze the importance of the relations and communications in the family during adolescence and their significance in the promotion of prosocial behavior in adolescents. Understanding adolescent-parent relationships, adolescent prosocial behavior, and sports participation as a means to promote positive educational interactions between parents and children and to promote prosocial actions, are essential.

## 2. Materials and Methods

### 2.1. Study Design

The researchers used mixed methods research [19,20], which is an approach to the collection, analysis, and integration of both quantitative and qualitative data of the research process within a single study [19]. The rationale for mixing both types of data is that neither quantitative nor qualitative methods are sufficient by themselves to capture the trends and details of situations, such as the complex issues of adolescents’ personal life experiences relative to their parents and their participation in youth sports.

**Figure 1:**
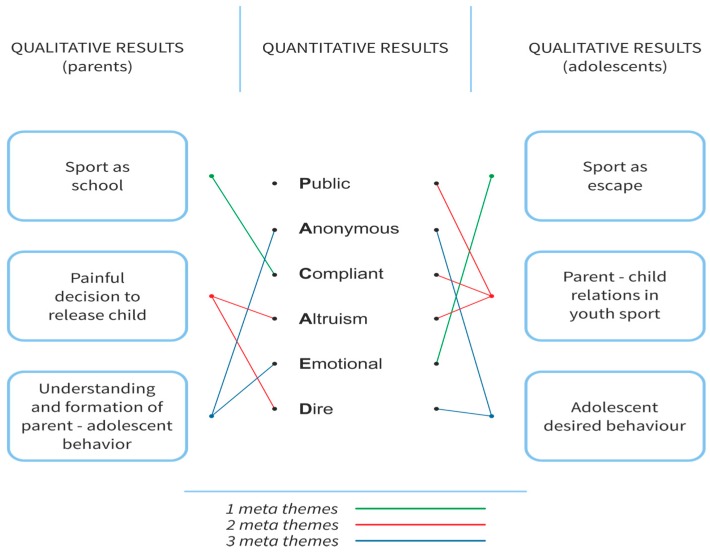
The integration of quantitative and qualitative results.

This study used an explanatory sequential mixed methods design that consisted of two distinct phases [19]. In this research design, the quantitative data is collected and analyzed first, followed by the collection and analysis of the qualitative data to explain or elaborate on the quantitative results obtained in the first phase. Therefore, the quantitative data and results provided a general picture of the research problem and the qualitative data and its analysis refined and explained the statistical results by exploring the participants’ perspectives in more depth. The quantitative and qualitative stages were connected [21]. We developed the interview protocol based on the results of the statistical tests in the first phase. Then we interviewed 24 participants (12 youth sports parents and 12 adolescent athletes) for a qualitative phenomenological interpretative analysis. The results of the quantitative and qualitative stages were integrated [21] in the discussion of the outcomes of the entire study (see Figure 1).

### 2.2. Quantitative Phase

#### 2.2.1. Participants and Procedures

Adolescents (716 girls and 632 boys) aged between 12 and 16 years (M = 14.2, SD = 1.51) participated in the quantitative study. We recruited from seven general education schools in Kaunas, Lithuania, using multi-stage sampling. In phase one we selected schools from all lists of schools. In the second step, we chose participants using a simple random sampling of classes from the selected schools. In the third phase, we invited all schoolchildren to participate in the survey from the selected classes. In total, 39.2% (*n* = 529) of all participants were involved in a competitive sport and had been playing sports for an average of 3.97 years (SD = 2.43).

We observed the human subject requirements of the first author’s institution. Before the research, we obtained permission from the school principal and parents to interview the children. We consented the children with parental approval. In some cases, we sought support from the regional Education Department before receiving the school principal’s approval. The researchers visited the schools at an arranged time. The students completed the survey with the researchers present in the classroom. The school’s social worker and the psychologist were on hand in the class. The lead researcher explained the study’s goal to the children and instructed them on how to fill in the questionnaire. We sealed all surveys in envelopes.

#### 2.2.2. Measures

For the quantitative phase, we utilized a cross-sectional survey design. To study the prosocial behavior of the schoolchildren, we used the revised prosocial tendencies measure (PTM-R) [22]. We translated PTM-R based on recommendations for adaptation of research instruments [23]. Several independent translations of the scale occurred. First, two English language professionals translated the statements on the scale into Lithuanian. Then those two translations were evaluated by a third English language professional, who recommended corrections, and the scales were corrected accordingly. Then two scientists working in the field of education and psychology evaluated the translated statements and performed the reverse translation. After translation procedures, we notified authors of the PTM-R and described the translation process. The authors reviewed and approved the translated PTM-R measure.

Reliability of the subscales ranged from 0.63 to 0.86. The PTM-R assesses six types of prosocial behaviors: public, anonymous, dire, emotional, compliant, and altruistic. Public prosocial behaviors are defined as behaviors intended to benefit others and enacted in the presence of others (three items; e.g., “I tend to help best when people are watching me”). Anonymous prosocial behaviors are defined as helping behaviors performed without the knowledge of who helped (four items; e.g., “I tend to help others in need when they do not know who helped them”). Dire prosocial behaviors are defined as helping in crisis or emergency situations (three items; e.g., “I tend to help people who are hurt badly”). Emotional prosocial behaviors are defined as an orientation toward helping others under emotionally evocative circumstances (five items; e.g., “I tend to help others especially when they are emotional”). Compliant prosocial behaviors are defined by helping others in response to a verbal or non-verbal request (two items; e.g., “When people ask me to help them, I do not hesitate”). Altruistic behaviors are defined as voluntary helping behaviors motivated primarily by concern for the needs and welfare of another (four items; e.g., “I feel that if I help someone, they should help me in the future”). The subjects rated each statement using a 5-point Likert-type scale (1 = “does not describe me at all”, 5 = “describes me greatly”, except for altruism, which used reverse scoring). Higher scores on each of the subscales reflected a stronger tendency to engage in prosocial behavior.

We assessed students’ sports participation using the question “Do you participate in competitive sports?” with two alternative answers: “Yes, I’m actively involved in sports, I go to sport classes at least two hours a week, participate in competitions and these activities have been lasting for at least two years”, “I exercise only in PE classes, I’m not physically active in my free time”.

#### 2.2.3. Data Analysis

The researchers performed the data analyses using SPSS version 22.0. (IBM, Chicago, IL, USA). We performed a MANOVA with the subscales as the dependent variables and group status (in athletics/not in athletics) as the categorical variable. We examined the univariate F-tests for each subscale. To determine meaningfulness of differences, we examined partial eta-squared (η^2^) and used Cohen’s [24] multivariate and univariate guidelines of 0.01 (small), 0.06 (moderate), and 0.14 (large).

### 2.3. Qualitative Phase

#### 2.3.1. Qualitative Research Design

An interpretative phenomenological analysis (IPA) was used [25]. IPA is concerned with the detailed examination of the human lived experience. IPA draws upon the fundamental principles of phenomenology, hermeneutics, and ideography. Phenomenological inquiry focuses on experiences within the consciousness of an individual.

#### 2.3.2. Participants and Procedures

The sample included 12 adolescent athletes and 12 youth sports parents who responded to the advertisement for volunteers. The youth were not participants in the quantitative study. All youths were 16 years of age with at least seven years of athletic sports experience. The parents’ average age was 49 years and they were highly educated. All of their children were approximately 16 years of age and had at least seven years of athletic experience. The parents and the adolescents were not familiar. We placed invitations in various clubs of different individual sports via ads. The participants were parents with child athletes at the time of the interview and athletes with at least four years of involvement in competitive sports.

The interview focused on exploring the “lived experiences” of the adolescents who participated in sports and their relationships with parents using an IPA. The explicit and thoughtful selection of participants occurred according to the study’s inclusion/exclusion criteria, which is consistent with the purpose of the study. We selected the survey participants based on the following selection criteria: homogeneity, information coverage and informed consent to participate in the survey [25].

#### 2.3.3. Interview Protocol Development

The questions given to the research participants focused on issues raised by the investigation. The protocol served only as a guide in each interview to prevent the researcher from uncontrolled deviation from the analyzed topic and to restrict free associations of the participants and the content of the narrative. Semi-structured interview questions began after the lead researcher established consent. Next, the participants shared more about themselves, their families and hobbies. Later, sensitive questions relating to the research emerged. The interviews concluded with a neutralizing inquiry about their feelings after the meeting and an opportunity to ask questions to the researcher.

#### 2.3.4. Data Collection

In this study, we collected the data on the experiences of youth sports parents and adolescents through semi-structured interviews. A semi-structured interview is a suitable method for data collection when the researcher’s priority is to understand the depth of the meaning. Therefore, the research focused primarily on insights and understanding. The interview questions were used as a suggestive guide rather than a mandatory program. Moreover, the lead researcher asked additional questions based on the individual stories and situations of a participant and the objectives set by the study. We treated the participants in the research as the actual research experts, and their proposed topics and suggestions were accepted and analyzed in depth.

#### 2.3.5. Data Analysis

The data analysis complied with the methodological requirements of IPA [25] and contained the following stages. The lead researcher audiotaped each interview and transcribed verbatim [20]. At this stage, the focus was on how the participants talked about themselves: their tone, rhythm, pauses, and changes in topics. IPA requires detailed and comprehensive interview transcription material (text), which is the object of the analysis. Therefore, we recorded some essential facts of participant interactions (laughing, crying, silence, apparent changes in mood, etc.). The first author transcribed the materials and the audio recordings obtained from 24 interviews (a total of more than 24 h) into text.

The researchers followed Yardley’s [26] four broad principles for the assessment of the quality of qualitative research: sensitivity to context; commitment and rigor; transparency and coherence; impact and importance. The research team secured the credibility of the findings by triangulating different sources of information, member checking, evaluating inter-coder agreement, rich descriptions of the cases, reviewing and resolving discontinuous evidence, and auditing by peers.

## 3. Results

### 3.1. Quantitative Phase

The MANOVA was significant, Pillai’s Trace = 0.018, F (61,353) = 4.08, *p* < 0.001, η^2^ = 0.02. Across the six subscales linear combination, the youth involved in athletics scored higher. The meaningfulness of difference in prosocial values was small but supported the expected trend to justify this study’s qualitative phase. Table 1 contains the univariate F-test results of prosocial behavior by participation in sports.

### 3.2. Qualitative Phase Adolescents’ Responses

In the qualitative follow-up phenomenology study analysis, three major themes emerged from the adolescents’ point of view (see Table 2): (1) sports as an escape; (2) parent-child relationships in youth sports; (3) adolescent desired behavior. Table 2 contains the theme table of athlete experiences. Text appearing in quotations “( )” are excerpts from the original text. The unique identifier in parentheses after the quote represents the interviewee’s pseudonym. In brackets “[ ]” we included additional information concerning the emotions, the manner of speaking, and the body movements of the research participants.

#### 3.2.1. Sports as an Escape

Adolescents see sports as an escape from their daily routine and the people around them. The interviews revealed adolescents’ feelings of being restricted, being put in a frame, and being under constant control at home, in the family, and at school. The adolescents complained that they could not express themselves in the way they wanted. Sports represent a private space for young athletes where they can escape from the surrounding world: “To me, sports are a kind of escape from the daily routine, like a release” (T5). “It’s a kind of discharge and I dedicate myself entirely to sports” (M1). “Getting away from routine, from school; you go away, you forget everything, you start communicating with different people in a different way; everything changes” (R2).

#### 3.2.2. Parent-Child Relationships in Youth Sports

Parent-child relationships depend not only on the activity itself but also on the building of interpersonal relationships at home: “They (parents) ask about new exercises that I did in the sports class or something of the kind. Perhaps they (parents) need this communication. But, if they need that and I don’t, it can’t be this way, this needs to be mutual; both parties should want to communicate” (T5).

Parents’ ability to verbalize and express their feelings, thoughts, and experiences and their ability not only to listen but also to hear their child’s message creates closeness and makes communication easier. Parents should also consider what is changeable internally to improve relationships with children. Adolescent athletes indicated that when parental behavior towards children changes, the quality of communication changes as well: “Parents should be observers I think and not interfere. They should be observers. Parents sometimes use inappropriate behavior: shouting and complaining with coaches and referees during the competition; such parent behavior is unwelcomed by athletes” (A12).

#### 3.2.3. Desired Behavior by Adolescents

The research revealed how adolescents’ learned behaviors in sports transfer into daily life, which is desirable to adolescent athletes.

##### Public

They wish to prove their worth, demonstrate their skills, and achieve the sought-after result: “My goal is to participate in competition, to show myself. I work hard for some important contest; all my goals are there, I want to achieve something and, of course, the main goal is to come, do your best, and win” (G4). “To demonstrate yourself, to show everyone what you achieved and knowing that you are better than your opponent is—the most important for every athlete” (A12).

##### Emotions

The emotions in athletes’ lives change dramatically and influence adolescents’ behavior: “The victory and defeat emotions, injuries, fear, and aggression follow every athlete who seeks the highest result” (D10). Despite a determined spirit, children’s moods reveal fear, which is indistinguishable from the excitement during preparation for the competition: “Before I go to a competition, of course, I feel fear, then I am afraid to lose’ (L6). ‘It’s ok to be a little bit afraid; you are more focused and concentrated, you don’t relax and lounge about; it is fun to feel a little tension” (R2).

##### Aggression

The aggression needed to achieve victory is the most prominent feeling in sports competitions. Anger is a driving force in many sports. Therefore, unsurprisingly, young athletes’ narratives reflected aggressive emotions about how they feel when entering a competition. Such sensations are not foreign to athletes, and once again, the interviews demonstrated adolescents’ surprising ability to enter another state of mind as learned through sports. In the interviews on other topics, the adolescents were gentle, romantic, and emotional, but in discussions about behavior, the adolescent athletes’ love of competition revealed an opposite ideology: “I just go feeling no pity because I go to end it” [smiles] (G4). “You go into competition, and you hate your opponent because it’s easier to fight this way and to win” (G4).

##### Prosocial Behavior

How young athletes can consciously leave stress and anger behind, control their emotions and behavior and follow the human values of athletic etiquette in such a short time after competitions is difficult to comprehend. “Respect for the opponent is always there; you shake hands and bow truly because that means respect” (G4). “Every athlete in one way or another is very sensitive towards his teammates, even the opponent: respect, help, and trust are always there” (A12).

Consequently, the emotions experienced in sports and behavior modeling transfer to everyday communication in life: “I learned a lot from sports, how to behave, how to communicate with people, family, and peers and how to make decisions and how to accept the right one” (R2). Therefore, not surprisingly, parent-child relationships reflect the strengthening of character in adolescent athletes. Sports influence the development of a purposeful, goal-seeking and robust personality. The understanding of common goals and objectives by both parents and children helps build the desired parent-child relationships in the family.

### 3.3. Qualitative Phase Parents’ Responses

In the qualitative follow-up, three major themes emerged from parents’ point of view (see Table 3): (1) sports as protection and as a school of life; (2) painful parental decisions to let their child act independently; (3) the understanding and forming of adolescents’ behavior through sports. Table 3 presents the theme table of parent experiences.

#### 3.3.1. Sport as a School of Life

All participants in the study, in one way or another, mentioned their child’s noticeable changes after involvement in sporting activities. Parents admit that sports encourage personality and adolescent behavior development in their growing child: “The importance lies not in physical abilities, but in thinking, in confidence; children who play sports are taught to see and understand the world differently, to behave in a different manner” (A1).

The parents see that the sporting environment helps foster their child’s individuality. Children develop a sense of duty, and their character becomes stronger with independence. Empathy, altruism, and prosocial behaviors develop during adolescence: “Children who are involved in sports are friendly, they share the last piece of a sandwich, the last coins” (D6) “It means that your child is emphatic. Other human values are very important. Her sense of duty has become stronger, and she is not afraid of work, she is ready to work hard and long, with patience to achieve the desired result [says slowly but surely]. She (daughter) achieved what she wanted through hard work” (M10).

It is a painful decision for parents to let their child act independently. The participants shared their regretful feelings that the participation of their developing children in sports had become a means of escaping the influence of parents. They understand that adolescence and detachment are inseparable. In this period of a child’s growth, parents must address new experiences: adolescents start rejecting the active participation of their parents in sports. Parents have to learn to equalize, to watch their child’s emotions, and adjust to an adolescent’s changing mood: “I haven’t seen her for a month again. I want to hug my child and so on, but I don’t have her. I became aware that I have no child, I am like some kind of supporting, invisible means used only for the financing of everything” (L12).

The youth expressed reticence and avoidance of parents tactfully and diplomatically: “He used to say: mom, you don’t need to come here [competitions], don’t worry. I asked him to call me, but he would answer: mom, I’m busy, I don’t have time” (C2).

#### 3.3.2. Understanding Adolescent-Parent Behavior in Youth Sports

##### Parents Model Their Behavior

Parents model their behavior and try to find the best ways of approaching their children. From the interviews with the parents, we observed that they all consider their input as significant and supportive, regardless of the form (positive and supporting or negative and destructive) and the level of their participation in their children’s sporting activities from their perspective. Every parent described different participation experiences, sometimes entirely unexpected. An agitated mother calls her situation desperate and her involvement in youth sports as “fake support” (D6). Another parent indicated careful parent involvement: “very carefully, everything must be very natural” (M10). Other types of parental involvement emerged: “over-involved parents” (A1), “critical parents” (S5), and “parents as coaches’ assistants” (Ch3). This topic emerged with the goal to describe parents’ understanding of support: what kind of support and behavior would be appropriate and what kind of support exists within their families.

##### Family Life Change

Looking back at the beginning of a child’s participation in sports, parents have interesting insights. The insights concern the fundamentals of a child’s education in a family that qualifies the understanding of support when the child starts playing sports: “Parents must be an example to be followed and communication with them must be pleasant; only then will children take on what you are trying to instill” (M10). Another research participant shares the same opinion: “first of all, there has to be an example in the family, especially for a small child, while he grows and becomes acquainted with the environment” (R2). Life changes a lot when children are born. Parents must satisfy their children’s needs and ignore some of their own needs. Chloe said that “life changed when they were born” (Ch3). Parents have new responsibilities and duties, and less spontaneity and enthusiasm remain, “you have to assume responsibility at once, you have to take care of things, you have to dedicate a big part of your time to something” (S5). The same is true for sporting activities. Children have different needs and expectations in new environments. New parental commitments must be established harmoniously with the child’s intentions in sports. New learning must occur, and everyone must adapt. For parents, this is similar to working with oneself. In sporting activities, parents learn to behave with children and learn to admit to their mistakes.

##### Parents Start Playing Sports Themselves

An instance that illustrates the changes in parents’ lives is at the time they follow their children’s example and start playing sports themselves. This example is a very positive behavior that shows that children’s involvement in sports brings positive changes to parents’ lives. Children help parents start playing sports: “When I took her to a sport class, I would sit on a bench and wait; then, I started exercising to make the waiting less boring” (M10). “It’s nice when children start playing sports, and then their parents get involved. They spend time together not only at home, but in the gym too. In competitions, parents start playing sports as well” (M10).

##### Parenting in Youth Sports Means Improving Themselves

New skills are required, perhaps different from those used at home, to show empathy when a child loses a competition: “sometimes, I have to express consolation. To me, this means improving my personality, learning what to say, when to speak, to not say too much nor too little; learning how to behave” (L12). The research participants believe that being in sports similarly to their child allows for the development of their personalities: “At the same time, I see the advantages for myself too because I am still learning to find balance in life [holds back tears], to choose priorities” (A1). “It teaches tolerance, patience, and believing in your child when no one else believes; I think that is the most important thing” [tries to hold back tears] (A1).

## 4. Discussion

The objective of this mixed method explanatory sequential study was to reveal the effects of participation in youth sports and relationships with parents on adolescents’ prosocial behavior. We identified six factors in the quantitative research (study I), including public, emotional, altruistic, dire, compliant, and anonymous behaviors, which predicted the prosocial behavior of adolescent athletes and non-athletes. During the quantitative study, we further analyzed whether a difference exists between adolescent athletes and non-athletes in their prosocial behaviors. The expression of prosocial behavior among adolescent athletes resulted in two scales (public and dire) compared to adolescents who do not play sports.

Next, we conducted a qualitative analysis (Study II) to understand the experience of athletes, to look at the study results from multiple forms of data and to gain a complete understanding of the research. Figure 1 illustrates the integration of the data from the mixed methods research (quantitative and qualitative). The joint display explicitly reflects the qualitative data complementing the six factors of the quantitative analysis, including public, emotional, altruistic, dire, compliant, and anonymous behaviors.

### 4.1. Public

In the quantitative research, the adolescent athletes most extensively expressed confidence in public prosocial behavior. Since public prosocial tendencies are behaviors that benefit others in front of an audience [11], the sporting environment as a publicly held space could explain the adolescents’ public prosocial behavior. Others (coaches, referees, teammates, peers, parents, siblings, fans) typically observe training, competitions, tournaments, and workouts. Perhaps this explains why adolescent athletes expressed higher scores on public prosocial behavior compared to non-athletes. The professionalization of sports has led adolescents to exhibit competitive behaviors in sports environments, which empowers public behavior in the long-term development of athletes in sporting environments and life. In the follow-up on the first qualitative study, adolescents’ responses to the theme “sports as an escape” revealed a general tendency among adolescents to understand how sports allow them to escape from the surrounding world and to show such a behavior publicly, willing everyone to understand them.

Also, we see parents’ experiences allow for the assertion that adolescents’ public prosocial behavior through theme “sport as protection and as a school of life” and the subtheme “a place to show themselves”. Parents described their children’s feelings of being proud to show their medals, trophies and other achieved victories and explained how much it means to them. The parents indicated that making these achievements visible to everyone was very important to the adolescent athletes.

### 4.2. Emotional

The quantitative results show that emotional prosocial behavior is not inherent to athletes. In the sports environment, emotional, altruistic, compliant prosocial actions may not be inherent in result-oriented athletes. Teenage athletes who are actively engaged in sports learn to set goals, plan their activities and seek desired results [27]. Athletes have different values, and their value orientations relate more to setting goals and creating career opportunities in the future compared to non-athletes. However, the qualitative results explain that emotional prosocial tendencies refer to actions in the context of emotionally rich circumstances. To athletes, the emotional prosocial behavior is seen through the theme “Desired behavior by adolescents” and the subtheme “emotions” which emphasizes athletes emotions experienced in a sporting environment, such as victory, defeat, injuries, fear, and aggression. The results suggest that athletes of middle-adolescent age express their emotional behavior in the sports competition environment. Carlo and Randall found that structure of prosocial behaviors is multidimensional in late adolescence age [28]. Researchers found that late adolescents who reported high levels of prosocial behaviors in emotional contexts (helping others under emotionally evocative circumstances), tended to be more responsible, demonstrated high levels of prosocial moral reasoning and strongly associated with sympathy responding and other-oriented personal tendencies. In contrast, adolescents who reported high levels of prosocial behaviors in front of others (public prosocial behaviors) demonstrated more hedonistic prosocial moral reasoning and were less sensitive towards others and reported lower perspective-taking tendencies.

### 4.3. Dire

The study revealed statistically significantly higher results for adolescent athletes’ dire prosocial behavior compared to non-athletes. Carlo and colleagues defined helping in emergencies as a dire prosocial tendency [10]. Sporting environments allow for the natural development of athletes’ character and understanding of ethical values not only in the context of sports but also in life [29]. We explored this phenomenon in more depth in the second qualitative study. The parents’ experiences of dire prosocial behavior are within the theme “the understanding and formation of adolescents’ behavior”, and its subthemes illustrate and explain how athletes help in emergencies. Children who are involved in sports are friendly, and they share the last piece of a sandwich and money.

### 4.4. Compliant

Quantitative research revealed that compliant behavior was not statistically significant in adolescent athletes. The qualitative theme “parent-child relationships in youth sport” from an adolescent point of view explains the results and demonstrates how compliant adolescent behavior develops and how parent-child relationships in youth sport appear. Adolescent athletes express themselves and illustrate how sport brings families closer together. Different communication between the parent and the child appears, with increased trust feeling and positive emotions that are very important to adolescent athletes. Compliant behavior is highly visible in parent-adolescent athletes. However, adolescents reflected their opinion about the need for mutual communication as well.

### 4.5. Altruistic

Altruistic behavior in adolescent athletes as shown in the quantitative results is not statistically significant. However, the qualitative phenomenological study explained and expanded upon the quantitative results. Parent interviews revealed that empathy, altruism, and prosocial behavior develop during adolescence, especially in sports environments. Parent interviews also highlighted that parenting style and family culture has a direct impact on adolescent altruistic behavior. This finding may be bi-directional. For instance, the study by [30] also examined the bidirectional relationships between authoritative parenting and adolescents’ prosocial behavior over a one-year period. The researchers emphasized the bidirectional relationships between parenting and prosocial behavior with a particular emphasis on the role of the adolescents’ prosocial behavior on subsequent parenting.

### 4.6. Anonymous

The results of the quantitative study show that adolescent athletes scored higher on public, dire and anonymous prosocial behavior. The qualitative phenomenological analysis showed how prosocial tendencies such as anonymous are inherent to athletes, especially relative to family involvement in youth sports. The responses provided by the adolescents indicate anonymous prosocial behaviors in the theme “desired behavior by adolescents”, which illustrates the different types of prosocial behaviors. Adolescent athletes’ anonymous prosocial behavior is reflected through the subtheme namely “prosocial behavior” (Table 1). Athletes emphasized that despite different forms of behavior, which occurs in the sporting environment, they highly express help, altruism, and empathy towards others and trying to act anonymously without demonstration. Prosocial development occurs within adolescent athletes without revealing itself explicitly. Instead, actions characterized by respect, trust and helpfulness in different situations towards teammates, family, peers, and even towards opponents reflect growth.

## 5. Conclusions

Adolescents’ relationships with parents are a critical factor and likely reflect the nature of the internal working models in youth sports that may function as a psychological template in the development of prosocial behavior during adolescence. Adolescents’ involvement in sports and their relationships with parents are significant predictors of prosocial behaviors. Public, anonymous and dire prosocial behaviors are evident in adolescent athletes. Maximizing emotional, compliant, altruistic prosocial behavior through fostering interpersonal relationships between a parent and a child is possible. This study expands upon the understanding of how the quality of parent-adolescent interpersonal relationships increases the development of adolescent prosocial behavior.

## Figures and Tables

**Table 1:** Results of adolescents prosocial behavior by athletes (*n* = 495) and non-athletes (*n* = 865).

Prosocial Behavior Scale	Athletes	Non-Athletes	F	η^2^
	Mean (SD)	Mean (SD)		
Public	3.14 (0.96)	2.92 (0.92)	16.34 ***	0.012
Anonymous	2.87 (1.04)	2.75 (1.00)	4.77 *	0.004
Dire	3.71 (0.88)	3.61 (0.85)	4.44 *	0.003
Emotional	3.66 (0.83)	3.64 (0.88)	0.34	0.000
Compliant	3.81 (0.90)	3.80 (0.85)	0.05	0.000
Altruistic	2.92 (0.91)	3.08 (0.90)	−9.97 ***	0.010

Note * *p* < 0.05; *** *p* < 0.001.

**Table 2:** The adolescent athletes’ themes.

Meta-Themes	Themes	Sub-Themes
Sports as an escape	Sport represents the personal space of young athletes	Sport is part of adolescents’ lives Spending time in another space, another world Knowing other people Adolescent’s acceptance by other people
	Sport as an excuse to escape from parental influence	It is mine and you cannot take it away from me The coach is the most important figure here
	Formation of adolescent’s identity through sports	Self-expression Comparison of self with others Finding personal authority Relationship between love for the chosen sport and self-identification
Parent-child relationships in youth sports	Sports bring families closer together	Different communication Secure relationships Trust Emotions
	Need for mutual communication	Conflicts in sports environments Need to respect adolescents’ opinions and choices Two-way communication system
	Parents are supportive in the event of failure or defeat in sports	Looking for warmth and condolence in the family Going through miseries together with the family Children and parents become closer in light of misfortunes
Desired behavior by adolescents	Public	Desire to be seen by others Demonstrate themselves Confidence Strive to be better than others
	Emotions	Victory Defeat Injuries Fear Aggression
	Aggression	Sport anger Aggressive attitudes in sports to achieve victory Winning ambition
	Prosocial behavior	Help Altruism Empathy

**Table 3:** Parent experiences in youth sports: Theme Table.

Meta-Themes	Themes	Sub-Themes
Sport as protection and as a school of life	Child’s preparation for life	Possibility to socialize Personality development Character traits Goal-seeking
	Sports as a way to prevent a child’s delinquent behavior	Purposeful activity Keeping the child away from negative influences A place to find new friends A place to show themselves
Parents’ painful decision to let their child act independently	Adolescence and detachment of the child	Disrespect to parental opinion Intentional opposition to parents Avoiding conversations
	Parent-child contradiction undermines relationships in the family	The notion that a teenager must listen to parents does not work Perception of helplessness is difficult to parents Opposing adolescents means losing them
	Letting the children go and act independently	Short-term detachment Watching from a distance Cautious approach
Understanding and formation of adolescents’ behavior through sports	Parents model their own behavior	Parental authority Fake support Careful involvement Parents as coaches Parents as consultants Parents as financial supporters
	Family life change	Life changes when a child is born Learning patience with the child Learning tolerance with the child Learning self-control
	Parents start playing sports	Children set examples to parents Parents get involved in sports
	Parenting in youth sports means improving oneself	Tolerance towards child Diplomatic behavior Empathy Pro-social behavior Altruism
